# Prevalence of complications associated with polymer-based alloplastic materials in nasal dorsal augmentation: a systematic review and meta-analysis

**DOI:** 10.1186/s40902-022-00344-8

**Published:** 2022-04-22

**Authors:** Seied Omid Keyhan, Shaqayeq Ramezanzade, Reza Golvardi Yazdi, Mohammad Amin Valipour, Hamid Reza Fallahi, Madjid Shakiba, Mahsa Aeinehvand

**Affiliations:** 1 Present Address: Maxillofacial Surgery & Implantology & Biomaterial Research Foundation, Tehran, Iran; 2 School of Dentistry, Ahvaz Jundishapur University of Medical Sciences, Ahvaz, Iran; 3 DDS,OMFS Founder & Director, Maxillofacial Surgery & Implantology & Biomaterial Research Foundation, Ahvaz, Iran; 4 Advanced Diagnostic and Interventional Radiology Research Center (ADIR), Tehran University of Medical Sciences, Tehran, Iran

**Keywords:** Rhinoplasty, Augmentation rhinoplasty, Alloplastic, Complications, Revision rate of rhinoplasty

## Abstract

**Background:**

Various techniques with different grafts and implants have been proposed to establish a smooth and symmetric nasal dorsum with adequate function. Broadly, two categories of materials have been used in this regard: alloplastic implant materials and autograft materials. The aim of these meta-analyses is to explore the incidence of complications after dorsum augmentation surgery using alloplastic materials.

**Materials and methods:**

After duplication removal 491 papers remained that title and abstract were assessed for eligibility. Regarding the study type, 27 observational studies were included, 21 retrospective and 6 prospective case series. A total of 3803 cases were enrolled in this systematic review and meta-analysis.

**Result:**

Twenty-seven articles reported on complications and outcomes of dorsal augmentation rhinoplasty with synthetic materials. In a random-effects model, the weighted mean percentage was 2.75% (95% CI 1.61 to 4.17%). the weighted mean percentage were 1.91% (95% CI 0.77 to 3.54%), 0.72% (95% CI 0.316 to 1.31%), and 0.78% (95% CI 0.43 to 1.24%) respectively.

**Conclusion:**

The widely used alloplasts were expanded polytetrafluoroethylene (ePTFE), high-density polyethylene, and silicone. The total rates for complications, infection, deviation, irregularity, hematoma, extrusion, and overcorrection were 2.75%, 1.91%, 0.72%, 0.70%, 0.78%, and 0.49%, respectively. The revision rate, based on the random effects model, was 6.40% with 95%CI (3.84 to 9.57).

**Trial registration:**

This meta-analysis was registered at the International Prospective Register of Systematic Reviews (PROSPERO, registration number CRD42020209644).

## Background

Over the time, the different approaches on rhinoplasty have shifted from reductive towards augmentative. The nasal dorsum height and shape, and its harmonious alignment with tip of nose, play a key role in creating perfect esthetic results [1, 2]. In cases with indistinct nasal bridges, dorsal deficiencies, and under-projected nasal dorsum, dorsal augmentation is the recommended procedure [3]. Various techniques with different grafts and implants have been proposed to establish a smooth and symmetric nasal dorsum with adequate function. Broadly, two categories of materials have been used in this regard: alloplastic implant materials and autograft materials [4].

First is widely used in west while the latter is the preferred item among Asian surgeons [5, 6].

There remains a controversy regarding the selection of the appropriate material with more advantages and lower complication rates. The autologous materials are preferred for dorsal augmentation due to low infection and extrusion rates and high biocompatibility. Although there remains concerns of complications such as major resorption and graft harvesting site morbidity with autologous grafting. Alloplastic materials such as silicone, ePTFE, and high-density polyethylene are an alternative. They are associated with varying incidences of infection and extrusion. Owing to their affordability, lack of any graft harvesting site and being tailorable to a particular deformity, in certain circumstances, alloplastic materials might be used [5]. In 2008, Peled et al. conducted a meta-analysis on rates of infection, extrusion, revision, and removal of different implants used in rhinoplasty surgery and mentioned that alloplastic implants have acceptable complication rates and might be used when facing limitations in using autogenous materials [7].

The aim of these meta-analyses is to explore the incidence of complications after dorsum augmentation surgery using alloplastic materials.

## Materials and methods

### Protocol and registration

This meta-analysis was registered at the International Prospective Register of Systematic Reviews (https://www.crd.york.ac.uk/ PROSPERO, registration number CRD42020209644). Also, the PRISMA 2020 Guidelines were followed in this systematic review and meta-analysis [8].

### PICO question

(P) Patient: patients with nasal dorsum deformities undergoing reconstructive or cosmetic rhinoplasty. (I) Intervention: reconstructive or cosmetic rhinoplasty of nasal dorsum augmentation without other nasal deformities. (C) Comparison: polymer-based alloplastic materials such as silicone, high-density polyethylene (Medpor), and polytetrafluoroethylene (Gore-Tex). (O) Outcome: complication rates including visible bulging of the graft, hematoma, graft displacement, irregularity, supra-tip depression, infection, deviation, overcorrection, insufficient augmentation, and major resorption.

### Search strategy

An electronic survey was conducted using the following databases up to and including September 2020 written in English without any time restriction: PubMed/MEDLINE, Google Scholar and the Cochrane Central Register of Controlled Trials (Central). The searching was completed by a manual hand search of the references of all selected full-text articles. The following search terms were screened with its appearance limited to title of the article: (a) “rhinoplasty,” (b) “nasal augmentation,” (c) “revisional rhinoplasty,” (d) “dorsum augmentation,” (e) “nasal dorsum,” (f) “alloplast,” (h) “silicone,” (i) “high-density polyethylene,” (j) “Medpor,” (k) “polytetrafluoroethylene,” and (l) “Gore-Tex” (Table 1).

**Table 1:** Search strategy

Search criteria	
PubMed (365)	((((((((alloplast[Title/Abstract]) OR (silicone[Title/Abstract])) OR (high-density polyethylene[Title/Abstract])) OR (Medpor[Title/Abstract])) OR (polytetrafluoroethylene[Title/Abstract])) OR (Gore-Tex[Title/Abstract])) AND (((((rhinoplasty[Title/Abstract]) OR (nasal augmentation[Title/Abstract])) OR (revisional rhinoplasty[Title/Abstract])) OR (dorsum augmentation[Title/Abstract])) OR (nasal dorsum[Title/Abstract]))
Google Scholar (172,11)	Concept 1: allintitle: “alloplast” OR “silicone” OR “high-density polyethylene” OR “Medpor” OR “polytetrafluoroethylene” OR “Gore-Tex” “nasal dorsum” Concept 2: allintitle: “alloplast” OR “silicone” OR “high-density polyethylene” OR “Medpor” OR “polytetrafluoroethylene” OR “Gore-Tex” “Rhinoplasty”
Cochrane library (27)	((alloplast) OR (silicone) OR (high-density polyethylene) OR (Medpor) OR (polytetrafluoroethylene) OR (Gore-Tex)) AND ((rhinoplasty) OR (nasal augmentation) OR (revisional rhinoplasty) OR(dorsum augmentation) OR (nasal dorsum))

### Study selection

Inclusion criteria were as follows:

1. Randomized clinical trials (RCTs), controlled clinical trials (CCTs), prospective and retrospective cohort studies, and case series with more than 10 participants which provided detailed report on complications (visible bulging of the graft, hematoma at the recipient area, graft displacement, irregularity, supra-tip depression, infection, deviation, overcorrection, insufficient augmentation, major resorption). (Report of at least one complication and revision surgery was mandatory.)
2. No follow-up restrictions
3. Only papers in English are included

Exclusion criteria were as follows:

1. Any cadaver studies or nonhuman studies
2. Studies reporting ratios (risk ratio, odds ratio, hazard ratio) instead of the absolute outcomes were not of our interest.
3. Any article that did not provide any detailed data regarding complication rates
4. Reports of using graft in other parts than nasal dorsum
5. Reports of using liquid alloplastic materials

### Data extraction

Based on a predefined paper checklist, the following data was retrieved from the finally included studies by two reviewers (M A.V and R.G) independently and supervised by third author (Sh.R). Any disagreements were resolved by discussion with a third author (Sh.R).

Data extraction included the following categories:

First author, year of publication, study location, study type, mean age, mean follow-up (range), sex, number of total cases, and cases with complication, incidence of complications after dorsum augmentation with polymer-based alloplastic materials such as silicone, high-density polyethylene (Medpor), and polytetrafluoroethylene (Gore-Tex), rates of complications, revision surgical procedures, and satisfaction rate (percent). The complications assessed were as follows:

visible bulging of the graft, hematoma at the recipient area, graft displacement, irregularity, supra-tip depression, infection, deviation, overcorrection, insufficient augmentation, major resorption.

### Risk of bias assessment within the studies

The methodological quality and synthesis of included materials was assessed using a tool for bias assessment in case series by Murad et al. [9]. There were 8 questions in the following domains: selection, ascertainment, causality, and reporting.

### Data analysis

Considering the challenges with meta-analysis in observational studies [10], we carefully checked whether included materials in hand were able to answer our clinical question (PICO). The proportion meta-analysis was performed using MedCalc version 18.9.1 (MedCalc Software Ltd., Ostend, Belgium). Both random and fixed model were used based on the heterogeneity. If the heterogeneity was significant, random model was preferred. We conducted the *χ*2 and *I*^2^ tests to convey the potential heterogeneity. Potential publication biases were evaluated using funnel plots.

## Results

### Study selection

**Fig. 1:**
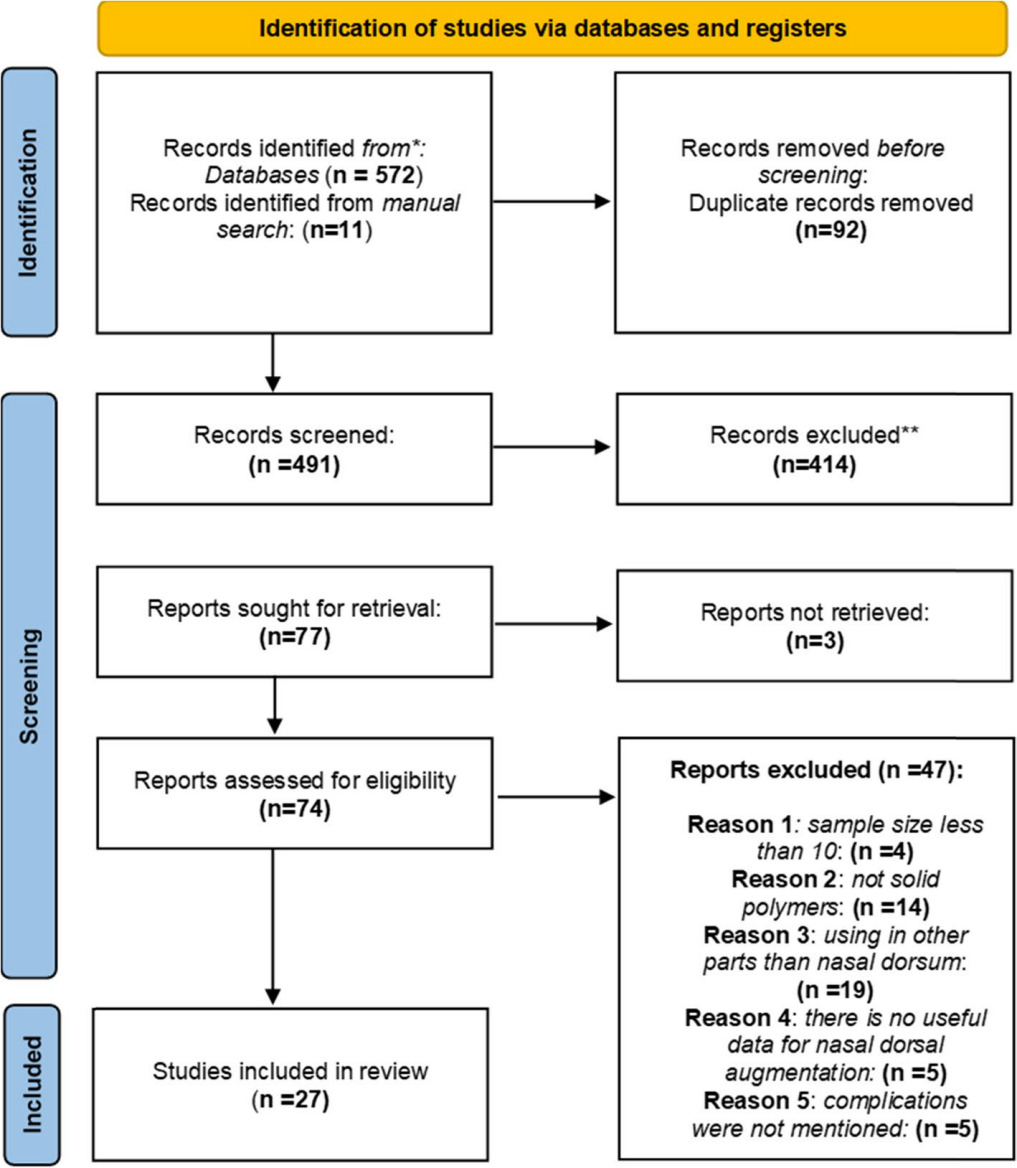
PRISMA Flowchart for study selection

Figure 1 shows the PRISMA flow diagram for the study selection process at different stages. 572 papers were obtained through the first search. After duplication removal, 491 papers remained that title and abstract were assessed for eligibility. Reports sought for retrieval of 77 papers. Of those, 50 papers were excluded with reason (3 reports not retrieved) and finally 27 papers remained which were included in the analysis [11–37].

### Study characteristics

The characteristics of included materials are shown in Table 2. Regarding the study type, 27 observational studies were included, 21 retrospective, and 6 prospective case series. A total of 3803 cases were enrolled in this systematic review and meta-analysis. The mean age of patients was 33 (age range 10–72). Although 3 papers did not specify mean and/or age range [16–19]. Four hundred twenty-eight cases were male and 2573 were female although 10 studies did not specify gender [13–32]. The mean follow-up time was 30 months with a range of 3 months to 15 years. Two papers did not report mean follow-up time specifically [16–19]. The included materials were conducted between years 1980 and 2019 in the following countries: South Korea [12–33], the USA [13–37], Spain [17], Taiwan [22–31], the UK [24], Turkey [26], Canada [27], Netherlands [28], China [34], Iran [29], Sweden [37], and Philippines [11].

**Table 2:** Characteristics of the included

Author (year), country of origin	Study type	Mean follow-up (range)	Number of cases (primary/secondary/tertiary)	Mean age/sex	Satisfaction
Yap, E. C.et al. (2011), [11] Philippines	**Retrospective**	Initial follow-up was on the fifth to seventh postoperative day. Successive follow-up visits occurred 2 weeks, 6 months, and 1 year after surgery	**1054 (1008 primary, 46 secondary)**	**34 (15 to 72)/955 female and 99 male**	**99.62%**
Kim, Y. S. et al. (2015) [12], Korea	**Retrospective**	9 to 108 months (mean 29.3 months)	**11 (all secondary)**	**37.1 years** **(8 female 3 male)**	**81.81%**
Scott Shadfar et al. (2015) [13], Pennsylvania	**Retrospective**	9(1–47)	**35 (23 primary, 12 secondary)**	**36 (17 to 65)/NR**	**Not mentioned**
Joo, Y. H. et al. (2016) [14], Republic of Korea.	**Retrospective**	12(4–115)	**176 (17 revision)**	**30.3 (11 to 69)/(96 male, 80 female)**	**75%**
Winkler, A. A. et al. (2012) [15] USA	**Retrospective**	12.1(0–74)	**75**	**46 (7 to 86)**	**Not mentioned**
Beekhuis, G. J.et al. (1980) [16], USA	**Retrospective**	NR	**30**	**NR**	**Not mentioned**
Alvarez-Buylla Blanco, M et al. (2011) [17], Spain	**Retrospective**	73(11–136)	**14(NR)**	**28 (16 to 50)/NR**	**Not mentioned**
Karnes, J et al. (2000) [18], USA	**Retrospective**	Up to 12 years	**30(NR)**	**NR**	**Not mentioned**
Colton, J. J. et al. (1992) [19], USA	**Retrospective**	NR	**93**	**NR**	**Not mentioned**
Niechajev, I (2012) [20], USA	**Retrospective**	6 months to 15 years (median, 7 years)	**52**	**18 to 70 years (median, 29)**	**90.56%**
Han (2012) [21] South Korea	Prospective/cohort	**20.9 months** **(2–105 months)**	**58** **48 primary, 10 secondary**	**29.4y(14–62 years)/5 male, 53 female**	**nm**
Chen (2010) [22] Taiwan	**Retrospective**	25.4 m (5–71)	**32**	**22 years (16–31)** **15 male, 17 female**	**90.6%** **84.4% patient satisfaction**
Hong et al. (2010) [23] South Korea	**Prospective/cohort**	34 months (12–98 months)	**873 total** **257 long follow-up and included**	**24 years (18–57)** **47 male, 826 female**	**nm**
Schwaiger et al. (2019) [24] UK	**Retrospective**	34.2 months (1–106 months)	**51 total case** **20 nasal dorsal**	**25.6 years (NM)** **24 male, 27 female**	**nm**
Jeong et al. (2018) [25] South Korea	**Retrospective**	6 months	**227**	**25 years (22–38 years)/21 male, 206 female**	**91.2%**
Turegun. M et al. (2008) [26] Turkey	**Prospective**	30 months	**14**	**35.5 (21–50)**	**100%**
Conrad. K et al. (2009) [27] Canada	**Retrospective**	71 months (1–17) years	**349**	**–** **(13–70) years**	**94.8%**
Lohuis. P.J.F.M et al. (2001) [28] Netherland	**Retrospective**	17.9 months (3–72) months	**66**	**35.9 years** **(10–66) years** **23 male and 44 female**	**Not mentioned**
Mohammadi Sh et al. (2014) [29], Iran	Prospective/cohort	3 years In monthly intervals	**38**	**36** **(15–58) years, 39 female and 25 male**	**Not mentioned**
Waldman S R et al. (1991) [30], USA	Retrospective	– (12–36) months	**17**	**33 years** **(17–48) years** **10 female and 7 male**	**94.1%**
Zelken Jonathan et al. [31] (2017), Taiwan	Retrospective	6 months (1–36) months	**177** **P: 63** **S: 144**	**34 years** **(19–72) years** **159 female and 18 male**	**4 unsatisfied**
Godin. M et al. [32] (1995), USA	Retrospective	25 months (6–80) months	**137** **P: 69** **S: 68**	**36 years** **(14–68) years** **Sex is not mentioned**	**100%** **All 137 patients**
Hwan Wang J et al. [33] (2007), Korea	Retrospective	31 months (12–39) months	**27** **P:23** **S: 4** **21 patients radix implant associated with dorsum**	**33 years** **(16–65) years** **15 female and 12 male**	**88.8%**
Zeng Yanjun et al. [34] (2002), China	Prospective	- 3 months–5 years	**98** **P: 92** **S: 6**	**–** **(17–49) years** **77 female and 21 male**	**63%**
Pham (2011) [35] USA	Retrospective	36 months	**23**	**20–57 years** **1 male** **22 female**	**1 not satisfied**
Pham and Hunter [36] (2006) USA	Retrospective	3 months–5 years	**19**	**18–56 years** **19 female** **0 male**	**1 not satisfied**
Niechajev [37] 1999 Sweden	Prospective	1–3 years	**23 dorsal**	**30 years (23–47 years)** **16 female** **11 male**	**nm**

*Abbreviations*: *nm* not mentioned

### Complications

The data on each complication are available in Table 3. The meta-analyses were available for the following complications: infection, deviation, irregularity, hematoma, extrusion, and over correction.

**Table 3:** Data regarding complications and revisions

Study, year of publication (number of cases)	Study design	Rates of complications							Revision
		Extrusion	Infection	Deviation (graft displacement)	Overcorrection	Hematoma	Irregularity	Others	
Yap, E. C. et al. [11] 2011 (*n* = 1054) **e-PTFE**	Retrospective	5	4	11	4	0	0	0	8
Turegun. M et al. [26] 2008, (*n* = 14) **Medpor**	Prospective	0	0	1	0	0	0	3 patients indicated that the difficulty of breathing started right after the surgery and 11 patients stated that it became worse.	1
Conrad. K et al [27] 2009, (*n* = 349) **Gore-Tex**	Retrospective	2	0	0	0	0	0	4 Soft tissue reaction	20
Lohuis. P.J.F.M et al. [28] 2001, (*n* = 66) **Gore-Tex**	Retrospective	0	0	0	1	0	0	0	1
Mohammadi Sh et al. [29] 2014, (*n* = 38) **Medpor**	Prospective/cohort	0	0	2	0	0	0	0	1
Waldman S R et al. [30] 1991, (*n* = 17) **Gore-Tex**	Retrospective	0	0	0	1	0	0	0	1
Zelken Jonathan et al. [38] 2016, (*n* = 177) **Composite (silicone-PTFE)**	Retrospective	0	2	8	0	4	0	0	12
Godin. M et al. [32] 1995, (*n* = 137) **Gore-Tex**	Retrospective	0	3	0	1	0	0	0	4
Hwan Wang J et al. [33] 2007, (*n* = 27) **silicone**	Retrospective	1	1	0	1	0	0	0	3
Zeng Yanjun et al. [34] 2002, (*n* = 98) **silicone**	Prospective	0	0	38	0	15	0	Drift o prosthesis Severe: 32 Mild: 43 Convexo-concave at nasal root Small: 19 Significant: 7 Small angle of the nose bridge less than 25°: 27	Nm
Hong et al. [23], 2010 (*n* = 257) **Gore-Tex**	Prospective/cohort	0	9	3	3	0	8	**Skin thinning 1/tip problems 9/ minor problems 4**/ 2 too low dorsum /	34
Schwaiger et al. [24] (2019) (*n* = 20) **Medpor**	Retrospective	0	1	2	0	0	0	0	Nm
Chen et al. [22]. 2010 (*n* = 32) **Medpor**	Retrospective	2	2	0	0	0	0	0	2
Han et al. [21]. 2012 (*n* = 58) **Medpor**	Prospective/cohort	0	2	0	0	0	0	0	2
Jeong et al. [25] (2018) (*n* = 227) **silicone**	Retrospective	2	5	4	0	0	7	**0**	7
Niechajev, I [20], 2012 (*n* = 53) Medpor	Retrospective	2	3	0	2	0	0	1 building 1 insufficient augmentation Patient dissatisfaction = 5	5
Colton, J.J et al. [19], 1992 (*n* = 93) Mersilene	Retrospective	0	8	0	0	0	0	0	4
Karnes Julie et al. 2000 [17] (*n* = 30) Medpor	Retrospective	2	0	0	0	0	0	0	2
Alvarez-Buylla Blanco, M et al. [17], 2011 (*n* = 14) Gore-Tex	Retrospective	0	4	0	0	0	0	0	3
Beekhuis, G. J.et al. [16] 1980 (*n* = 30) Polyamide	Retrospective	0	3	0	0	0	0	0	3
Winkler, A. A. et al. [15], 2012 (*n* = 75) ePTFE	Retrospective	2	4	0	0	0	0	0	nm
Joo, Y. H. et al. [14], 2016 (*n* = 176) ePTFE	Retrospective	0	1	2	0	0	1	Obvious implant contour = 2 Short nose deformity = 1	nm
Scott Shadfar et al. [13], 2015 (*n* = 35) ePTFE	Retrospective	1	1	0	0	0	1	0	2
Kim, Y. S. et al. [12] 2015 (*n* = 11) 8 silicone 3 Gore-Tex	Retrospective	0	1	0	0	0	0	0	2
Pham and Hunter [36] 2006 (*n* = 19) **Medpor**	Retrospective	0	0	0	0	0	0	0	1
Pham [35] 2011 (*n* = 23) **Medpor**	Retrospective	0	0	0	0	0	0	0	1
Niechajev [37] 1999 **Medpor (*n*= 23)**	Prospective	1	1	0	0	0	1	2 manageable complications	2

**Fig. 2:**
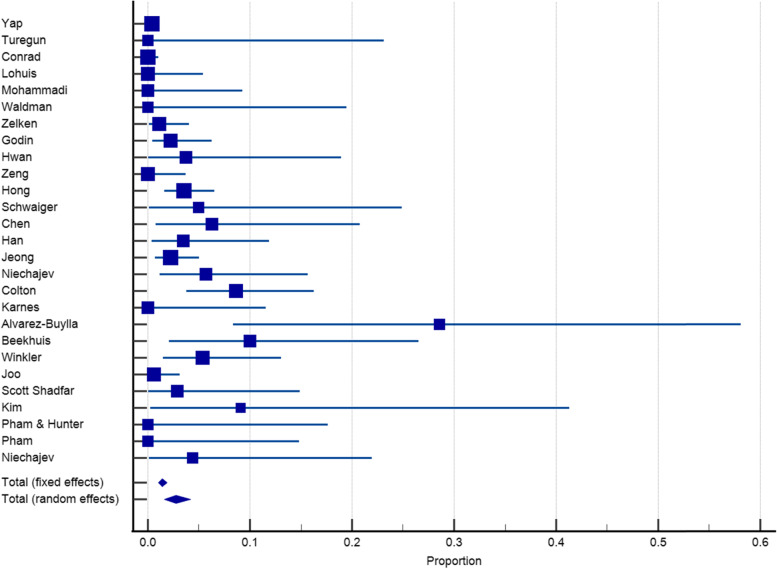
The weighted mean percentage of Infection rates reported synthetic materials in both fixed and random-effects

**Fig. 3:**
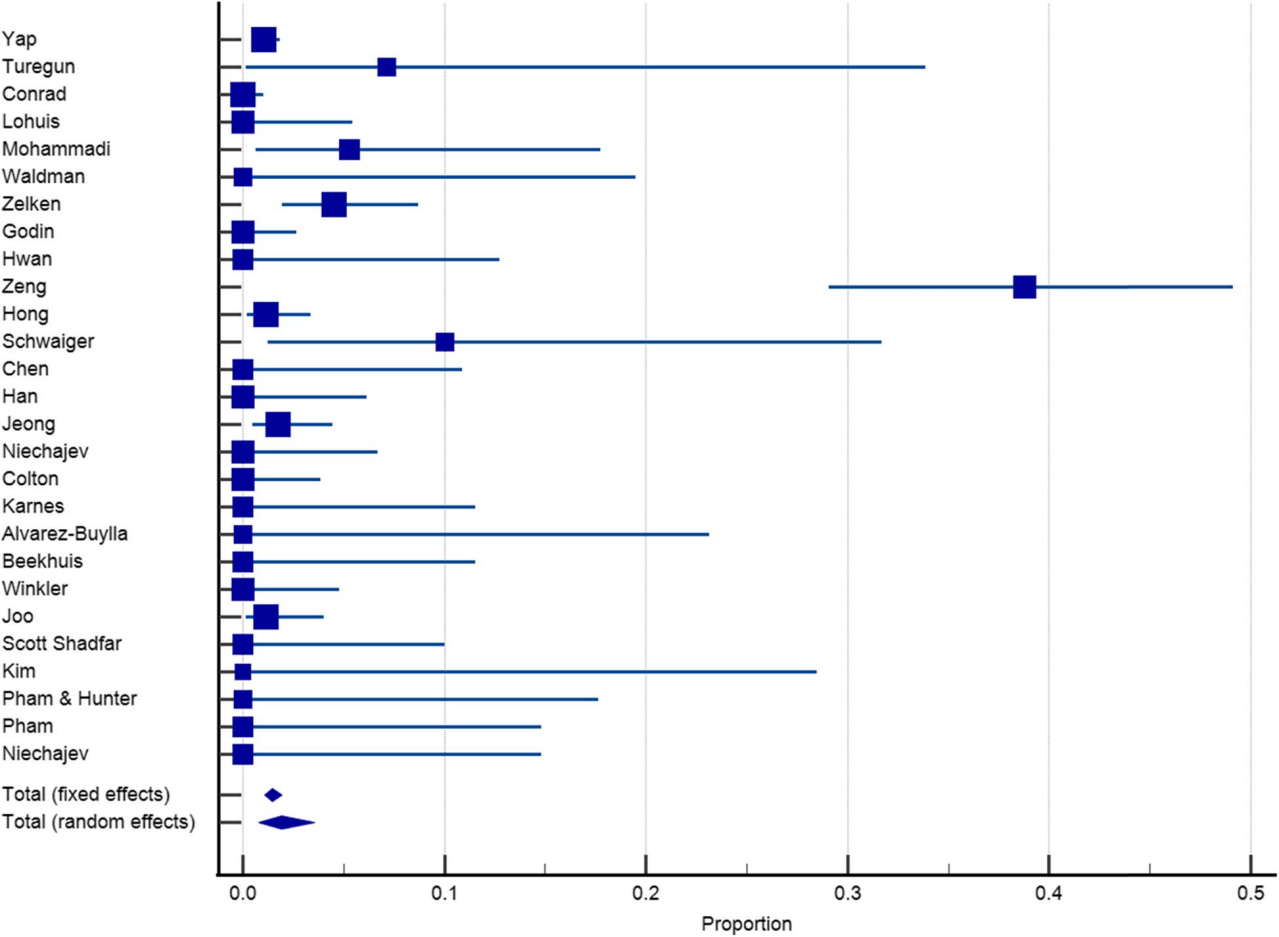
Deviation rates reported for synthetic materials in both fixed and random-effects model

**Fig. 4:**
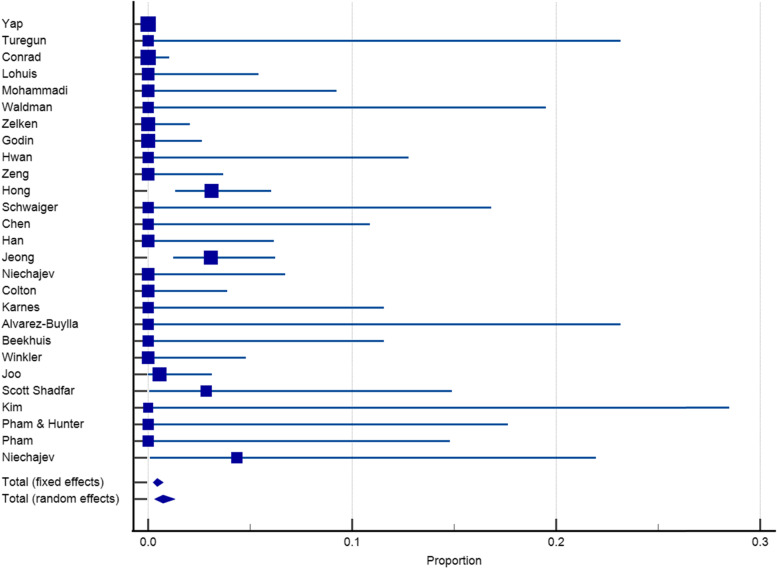
Irregularity rates reported for synthetic materials in both fixed and random-effects model

**Fig. 5:**
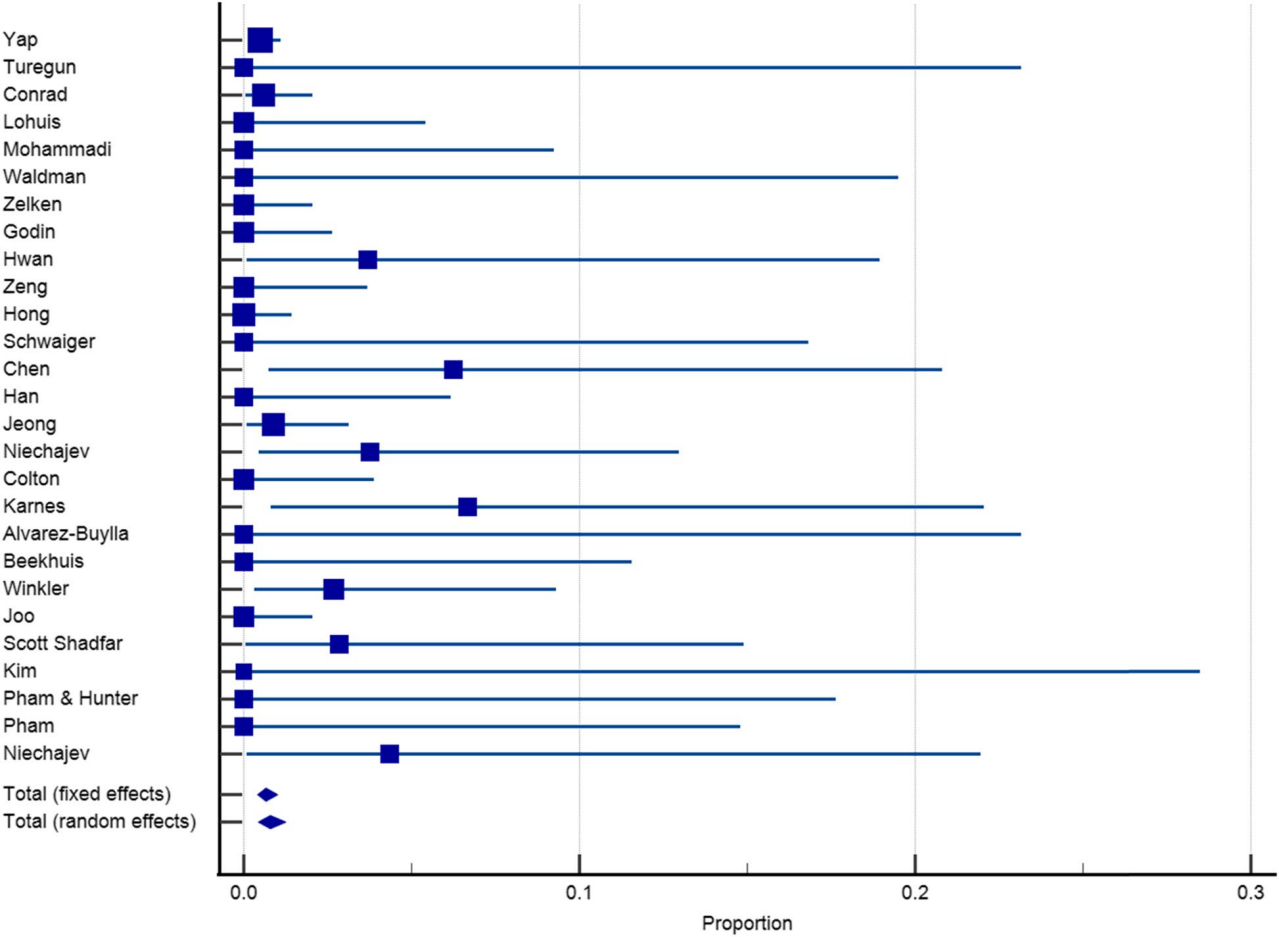
Extrusion rates reported for synthetic materials in both fixed and random-effects model

**Fig. 6:**
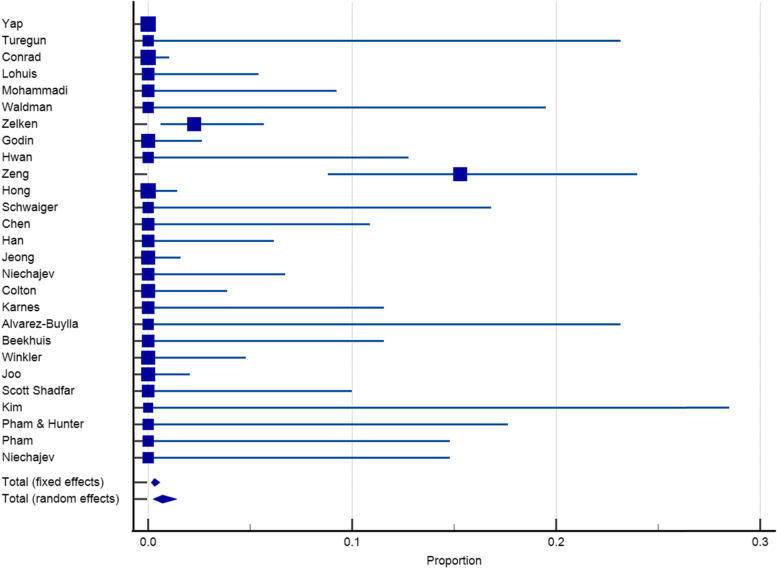
Hematoma rates reported for synthetic materials in both fixed and random-effects model

**Fig. 7:**
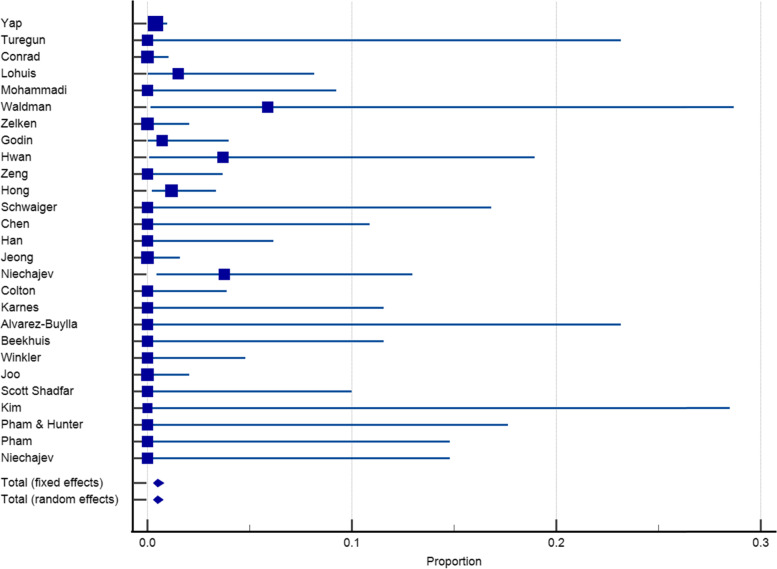
Over correction rates reported for synthetic materials in both fixed and random-effects model

Twenty-seven articles with a sample size of 3153 reported on the incidence of infection after dorsum augmentation with synthetic materials. In a random-effects model, the weighted mean percentage was 2.75% (95% CI 1.61 to 4.17%) (Fig. 2). The same articles (3153 cases) also reported on the deviation and irregularity and extrusion rates; the weighted mean percentage were 1.91% (95% CI 0.77 to 3.54%) (Fig. 3), 0.72% (95% CI 0.316 to 1.31%) (Fig. 4), and 0.78% (95% CI 0.43 to 1.24%) (Fig. 5) respectively. The weighted mean of hematoma and over-correction in a random-effects model were 0.70% (95% CI 0.24 to 1.40%) and 0.49% (95% CI 0.28 to 0.77%) respectively (Figs. 6 and 7).

### Other complications

Some rare complications did not meet the criteria for meta-analysis and therefore reported narratively:

One case of opening of the tube the diced cartilage pieces, in a cleft lip patient, pleural tear, and air leak during rib harvesting, of strike skin necrosis (Table 3)

### Revision rates

**Fig. 8:**
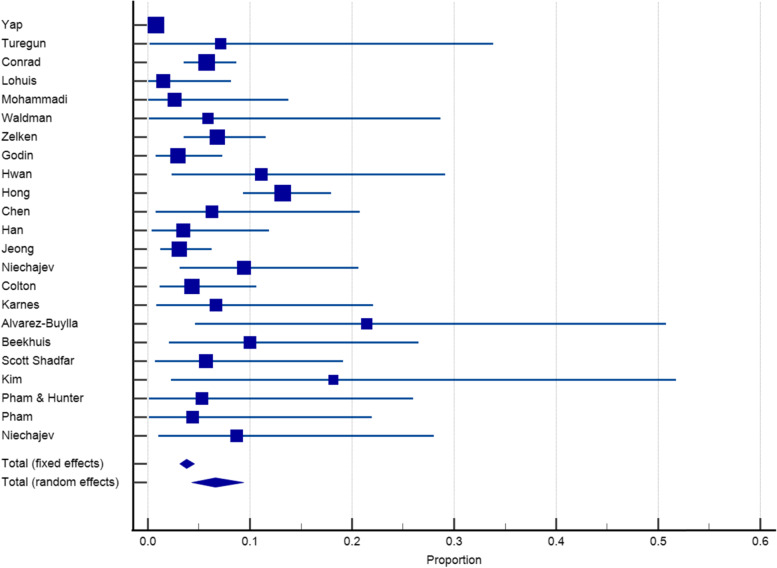
Revision rates reported for synthetic materials in both fixed and random-effects model

**Fig. 9:**
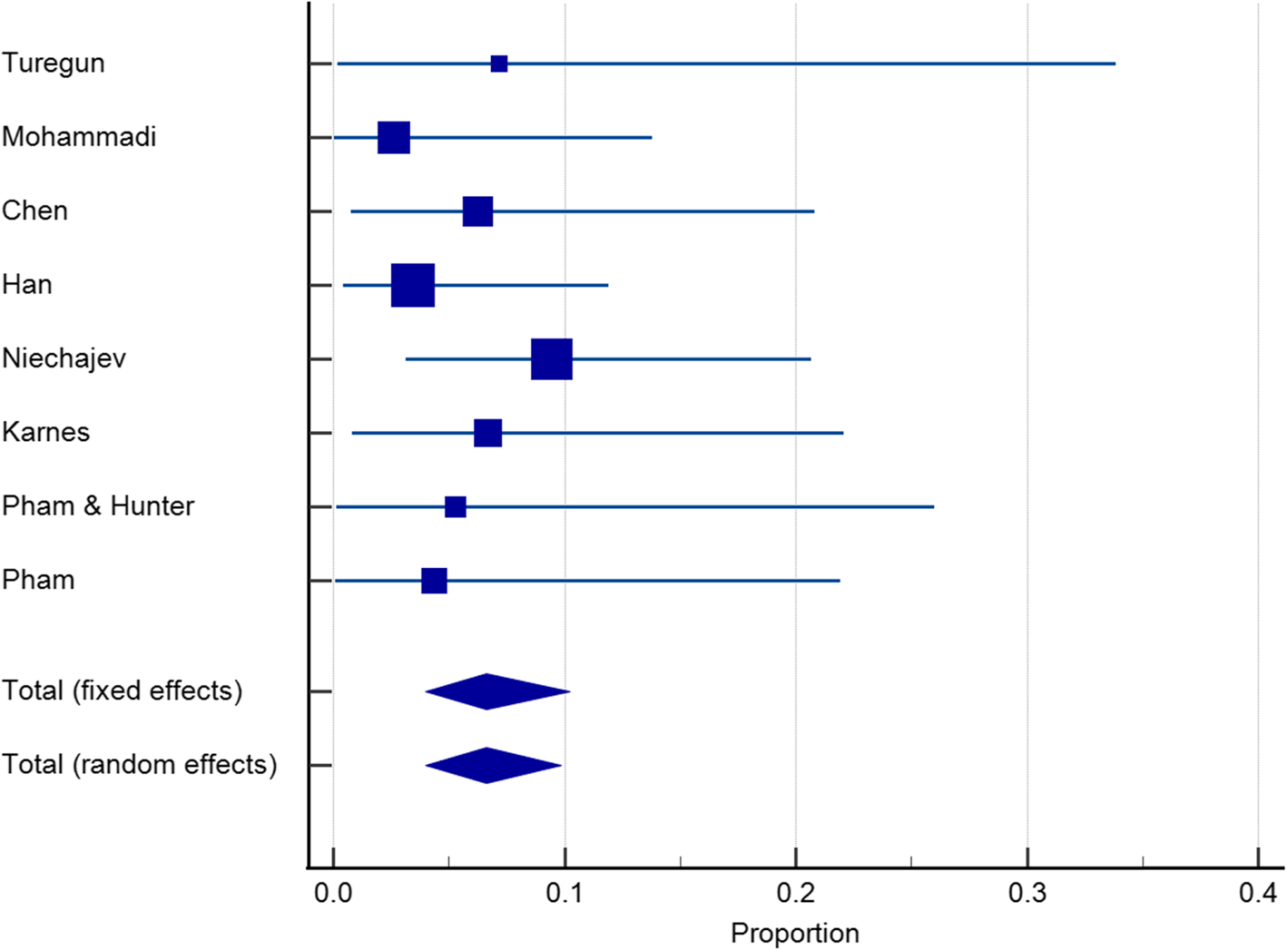
Revision rates reported for Medpore in both fixed and random-effects model

**Fig. 10:**
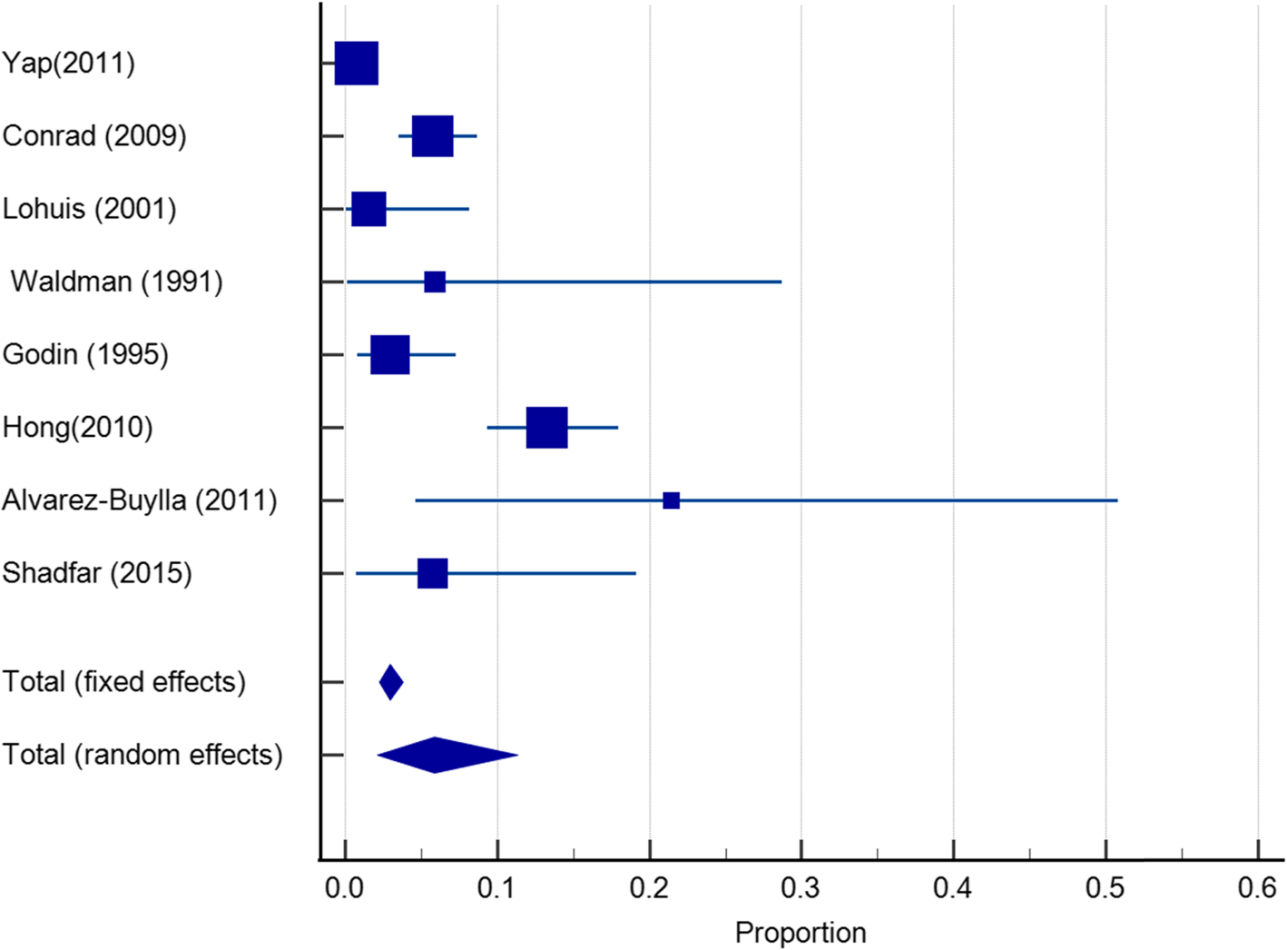
Revision rates reported for Gore-Tex in both fixed and random-effects model

**Fig. 11:**
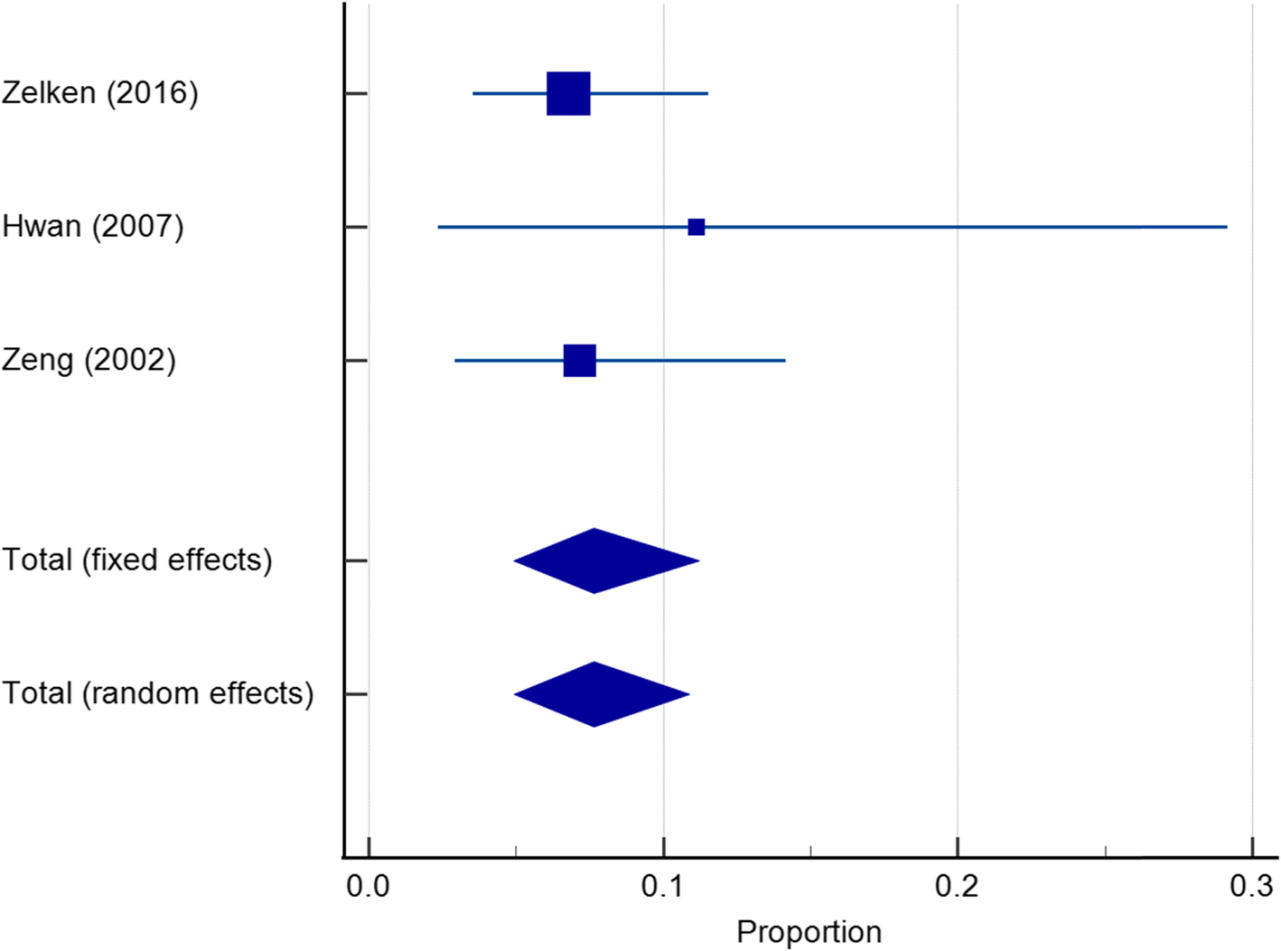
Revision rates reported for silicone in both fixed and random-effects model

All included material with a total of 451 patients reported on revision surgery rates; the pooled rate was 6.40% (95% CI 3.81 to 9.57%) (Fig. 8). Four papers did not report a specific number of revision surgery and therefore not included in the meta-analysis. The revision rates for the three most commonly used materials (Medpore, Gore-Tex, and silicone) were 6.61% (95% CI 3.98 to 9.85%), 4.91% (95% CI 1.81 to 9.43%), and 7.64% (95% CI 4.93 to 10.88%) respectively (Figs. 9, 10, and 11).

### Publication bias

We performed funnel plot for publication bias assessment for each of variables. In the current study, some levels of bias were reported for all complications.

## Discussion

One of the greatest challenges in rhinoplastic surgeries is the management of nasal dorsum augmentation. Due to the ease of use, producing ideal aesthetic results and removing needed for graft harvesting sites, alloplastic materials play an important role in typical cosmetic dorsal augmentation [1]. The use of alloplastic materials to address dorsal deficiencies is common among patients avoiding autogenous tissue harvest. Also, patients with several prior nasal operations and significant deformities are the best candidates for alloplastic materials as they mostly have depleted potential autogenous harvesting site [7]. Although there are concerns over several complications associated with synthetic materials including infection, deviation, extrusion, etc. [1]. In this systematic review and meta-analysis, we determined the complications reported for different alloplastic materials. Twenty-seven articles reported on complications and outcomes of dorsal augmentation rhinoplasty with synthetic materials. The widely used alloplasts were expanded polytetrafluoroethylene (ePTFE), high-density polyethylene, and silicone. The total rates for complications, infection, deviation, irregularity, hematoma, extrusion, and over correction were 2.75%, 1.91%, 0.72%, 0.70%, 0.78%, and 0.49%, respectively. The revision rate, based on random effects model, was 6.40% with 95% CI (3.84 to 9.57).

We reported a subsequent revision of 0–21% in our included studies. The pooled rate for the need for revision surgery was 6.40%. The revision rates for the three most commonly used materials Med-pore, ePTFE, and silicone were 6.61%, 7.06%, and 7.64%, respectively. The decision for implant removal is quite controversial; although, surgical removal of infected implants followed by an immediate or delayed reconstruction has higher chances of resolution [39].

The highest revision rates were related to silicone (7.64%). A similar study reported 6.5% revision rate for silicone implants [7]. Being the most commonly used alloplastic material in Asian countries, silicone is a smooth, cost benefit, and easy-carved implant which can be easily removed in case of failure. The lack of pores leads to fibrous capsule formation around the implant within the body.

Infections and displacement are the main causes of revision surgery in silicones and therefore in order to reduce such problems aggressive modification of the natural barriers and anatomical structure should be strictly avoided [40]. If shaped appropriately according to the nasal phenotype, the extrusion rate would reduce [41]. To manage and reduce complications, this method supports alloplastic materials better for patients with thicker skin than for patients with thinner skin.

The high-density polyethylene (Medpore), with pore size range from 160 to 368 μm, and more than half of these pores are larger than 150 μm in diameter and have excellent biocompatibility. In candidates of augmentation rhinoplasty with severe over resections or severe deformities, these implants have been a useful option. Our findings for revision surgery of high-density polyethylene have been higher than previously reported rates [7].

The revision rate for polytetrafluoroethylene/expanded polytetrafluoroethylene (Gore-Tex) was 4.91%. This hydrophobic polymer with pores of up to 30 μm allows for bacterial adherence and levels of issue integration that provides implant stability with ease of removal if needed. Our results are in line with previous studies mentioning low incidence of revision rates compared with other synthetic materials. A similar previous meta-analysis (in 2008) reported the removal of 3.1% for both ePTFE and high-density polyethylene [7].

Nevertheless, our results suggested a relatively high total rate for revision rate (6.40%) compared with autogenous grafts (3.03%) [42]. This might be attributed to the fact that infection in synthetic materials, unlike autogenous grafts, conservative treatments are inapplicable and mostly require revisional surgery [42, 43]

The use of autograft materials in nasal dorsum augmentation is a safer treatment with fewer complications compared to the alloplastic method. Complications of using autogenous grafts materials such as diced cartilage include graft resorption, insufficient augmentation, deviation (graft displacement), infection, irregularity, supra-tip depression, over-correction, hematoma at the recipient site, and the visible bulging of the graft. According to the findings of the article, infections caused by the use of alloplastic usually require revision surgery, while most infections that occur in the autograft method can be controlled by intravenous antibiotics [42].

In fact, it can be said that the complications of the autograft method are manageable and controllable complications.

Also, the use of alloplastic materials is a risky method in comparison with autograft materials taken from the patient himself, because the use of alloplastic materials acts as a foreign body in the body and its high-risk side effects can lead to nasal deformity and aesthetic complications. Undesirability in the systematic study [44] was reported in autograft materials, which are usually removed from the abdomen or thighs, has fewer reported complications after surgery, and in most cases, complications such as numbness gradually decrease after surgery and are completely eliminated by 3 months after surgery.

In addition to the side effects of using alloplastic materials, some side effects may be preventable, such as bending along the natural convexity, bone resorption, and foreign body reactions such as fibrous capsule formation and tissue ingrowth.

Due to the fact that the complications mentioned throughout the article are not only common but also lead to major problems both during surgery and after surgery. By examining the problems and complications of this method, treatment-related techniques will be developed in the future. With the passage of time and the development of new surgical methods and materials, it shows that current methods are always associated with complications, and at no time are failures and complications announced at the same time as successes.

Current articles widely support autografts instead of using alloplastic in rhinoplasty. Surgeons describe alloplastic implants as dangerous, unpredictable, and hard to use. Therefore, the reported complications are less than 5%. Complications that require revision surgery and cause the material to be removed are 3.7%, which is a significant amount compared to the use of autografts [45], which is 1% [96]. Therefore, the use of alloplastic materials seems to be mentioned with the desire of the patient and the surgeon and acceptance of the possibility of complications (Tables 4 and 5).

**Table 4:** Excluded studies with reason

Title (reference)	Reason on exclusion
1. Medpor in maxillofacial deformities: report of three cases [46]	3 cases
2. A case report of ophthalmic artery emboli secondary to calcium hydroxylapatite filler injection for nose augmentation–long-term outcome [47]	Not solid polymers
3. Case reports of adipose-derived stem cell therapy for nasal skin necrosis after filler injection [48]	Not solid polymers
4. Complete septal extension grafts using porous high-density polyethylene sheets for the westernization of the Asian nose. [21]	Not for dorsum
5. Retinal branch artery embolization following hyaluronic acid injection: a case report [49]	Not solid polymers
6. Two cases of adverse reactions of hyaluronic acid-based filler injections [50]	Not solid polymers
7. A newly designed minigraft to achieve angularity and projection of the nasal tip: the asymmetrical bipyramidal graft [51]	Not dorsum separately
8. Foreign body reaction to Radiesse: 2 cases [52]	2 cases
9. Plastic surgery for women [53]	Not related
10. Midline volume filler injection for facial rejuvenation and contouring in Asians [54]	Not solid polymers
11. Non-surgical rhinoplasty with hyaluronic acid fillers: predictable results using software for the evaluation of nasal angles [55]	Not solid polymers
12. Nasal filling in plastic surgery practice: primary nasal filling, nasal filling for post-rhinoplasty defects, rhinoplasty after hyaluronidase injection in dissatisfied nasal filling patients [56]	Not solid polymers
13. Calcium hydroxylapatite gel (Radiesse) injection for the correction of postrhinoplasty contour deficiencies and asymmetries [57]	Not dorsum separately
14. Augmentation rhinoplasty: observations on 1200 cases [58]	Not solid polymers
15. Secondary rhinoplasty of the Asian nose: correction of the contracted nose [59]	Not solid polymers
16. Revision rhinoplasty in ethnic patients: pollybeak deformity and persistent bulbous tip [60]	Not dorsum separately
17. Correction of the supratip deformity of the nose [61]	Not dorsum separately
18. Assessment of nostril symmetry after primary cleft rhinoplasty in patients with complete unilateral cleft lip and palate [62]	Complications were not assessed
19. Operative techniques in Asian rhinoplasty [63]	Operative techniques
20. E-M shaped septal encircling with Medpor reconstruction on crooked noses: personal technique and postoperative results [64]	Septal encircling reconstruction, not related
21. Late complications of nasal augmentation using silicone implants [65]	Complications were not mentioned
22. Periorbital necrotizing fasciitis and orbital apex syndrome as a delayed but emergent complication of silicone nasal augmentation [66]	Case report
23. Management of wide nasofrontal angle with GORE-TEX implants [67]	Not dorsum
24. Silicone rubber implants in nasal reconstructive surgery [68]	Not dorsum separately
25. Availability and safety of osteotomy in esthetic rhinoplasty of east Asian patients [25]	Not dorsum separately
[69] Evaluation and proportion in nasal filling with hyaluronic acid	Not found yet. Searching (not retrieved)
27. Prevention and management of iatrogenic blindness associated with aesthetical filler injections [70]	Not related
[71] Efficacy and safety of a hyaluronic acid filler to correct aesthetically detracting or deficient features of the Asian nose: a prospective, open-label, long-term study	Not solid polymers
29. Application of a porous polyethylene spreader graft for nasal lengthening in Asian patients [72]	Not dorsum separately
30. Use of fillers in rhinoplasty [73]	Not solid polymers
31. Simple implant augmentation rhinoplasty [74]	No number
32. Soft and firm alloplastic implants in rhinoplasty: why, when and how to use them: a review of 311 cases [75]	Not dorsum separately
33. The use of expanded polytetrafluoroethylene in short nose elongation: fourteen years of clinical experience [76]	L shaped
34. A novel method to enhance dynamic rhinoplasty outcomes: double “V” carving for alloplastic grafts [77]	L-shaped
35. The nonsurgical rhinoplasty: a retrospective review of 5000 treatments [78]	Not dorsum separately
36. The use of Medpor implants for midface contouring in cleft patients [24]	Not dorsum separately
37. Long-term results of high-density porous polyethylene implants in facial skeletal augmentation: an Indian perspective [79]	Not dorsum separately
38. ^a^Are polytetrafluoroethylene (Gore-Tex) implants an alternative material for nasal dorsal augmentation in Asians? [23]	Not dorsum separately
39. Nasal dorsum reconstruction with alloplastic material [80]	Complications were not mentioned
40. Injection rhinoplasty with hyaluronic acid and calcium hydroxyapatite: a retrospective survey investigating outcome and complication rates [81]	3 cases
41. Use of porous high-density polyethylene in revision rhinoplasty and in the platyrrhine nose [82]	Not dorsum separately
42. Soft tissue fillers in the nose [83]	*Not solid polymers*
43. Problems associated with alloplastic materials in rhinoplasty [84]	Complications not mentioned
44. [Nasal dorsal augmentation] [85]	Not in English (not retrieved)
45. Rhinofilling with hyaluronic acid thought as a cartilage graft [86]	*Not solid polymers*
46. A simple technique for the correction of maxillonasal dysplasia using customized expanded polytetrafluoroethylene (ePTFE) implants [87]	“L”-shaped ePTFE
47. Revision rhinoplasty of Asian noses: analysis and treatment [88]	Complications not mentioned
48. [Pyodermatitis of the nasal pyramid disclosing a complication of rhinoplasty with silicone implant] [89]	Not in English (not retrieved)
49. Use of porous high-density polyethylene in revision rhinoplasty and in the platyrrhine nose (Romo III et al.) [82]	68 patients had dorsum tip implants/complications were not categorized
50. Nonsurgical rhinoplasty with the novel hyaluronic acid filler VYC-25L: results using a nasal grid approach (Bertossi et al.) [90]	*Not solid polymers*

**Table 5:** Risk of bias assessment

	1	2	3	4	5	6	7	8	Total
Yap, E. C.et al. (2011), [11] Philippines	y	y	Y	NA	NA	NA	Y	y	5/8
Kim, Y. S. et al. (2015) [12], Korea	y	y	y	NA	NA	NA	Y	y	5/8
Scott Shadfar et al. (2015) [13], Pennsylvania	y	y	Y	NA	NA	NA	Y	y	5/8
Joo, Y. H. et al. (2016) [14], Republic of Korea.	y	y	y	NA	NA	NA	Y	y	5/8
Winkler, A. A. et al. (2012) [15] USA.	y	y	Y	NA	NA	NA	Y	y	5/8
Beekhuis, G. J.et al. (1980) [16], USA	y	y	y	NA	NA	NA	N	y	4/8
Alvarez-Buylla Blanco, M et al. (2011) [17], Spain	y	y	Y	NA	NA	NA	Y	y	5/8
Karnes, J et al. (2000) [18], USA	y	y	y	NA	NA	NA	Y	y	5/8
Colton, J. J. et al. (1992) [19], USA	y	y	Y	NA	NA	NA	N	y	4/8
Niechajev, I (2012) [20], USA	y	y	y	NA	NA	NA	Y	y	5/8
Han (2012) [21] South Korea	y	y	Y	NA	NA	NA	Y	y	5/8
Chen (2010) [22] Taiwan	y	y	y	NA	NA	NA	Y	y	5/8
Hong et al. (2010) [23] South Korea	y	y	y	NA	NA	NA	Y	y	5/8
Schwaiger et al. (2019) [24] UK	y	y	Y	NA	NA	NA	Y	y	5/8
Jeong et al. (2018) [25] South Korea	y	y	y	NA	NA	NA	Y	y	5/8
Turegun. M et al. (2008) [26] Turkey	y	y	Y	NA	NA	NA	Y	y	5/8
Conrad. K et al. (2009) [27] Canada	y	y	y	NA	NA	NA	Y	y	5/8
Lohuis. P.J.F.M et al. (2001) [28] Netherland	y	y	Y	NA	NA	NA	Y	y	5/8
Chen Liang et al. (2014) [91] China	y	y	y	NA	NA	NA	Y	y	5/8
Mohammadi Sh et al. (2014) [29] Iran	y	y	Y	NA	NA	NA	Y	y	5/8
Waldman S R et al. (1991) [30] USA	y	y	y	NA	NA	NA	Y	y	5/8
Zelken Jonathan et al. [31] (2017) Taiwan	y	y	Y	NA	NA	NA	Y	y	5/8
Godin. M et al. [32] (1995) USA	y	y	Y	NA	NA	NA	Y	y	5/8
Hwan Wang J et al. [33] (2007) – Korea	y	y	y	NA	NA	NA	Y	y	5/8
Zeng Yanjun et al. [34] (2002) China	y	y	Y	NA	NA	NA	Y	y	5/8
Pham (2011) [35] USA	y	y	y	NA	NA	NA	Y	y	5/8
Pham and Hunter [36] (2006) USA	y	y	Y	NA	NA	NA	Y	y	5/8
Niechajev [37] 1999 Sweden	y	y	y	NA	NA	NA	Y	y	5/8

## Limitations and strengths

Reports of complications often come from other investigators, citing their own experience with implants inserted by other surgeons. The major limitation with current systematic review and meta-analysis was the descriptive nature of much of the current literature and lacking comparator groups. Also, a proper tool for quality assessment in case series was lacking and we had to make adaptations in domains. Some levels of bias might be caused by excluding non-English materials.

The time frame for follow-ups in this study assumed rationale for complications appearance. Having a clear understanding of complications of each material and the ways to prevent and treat them is possible by accurate disclosure of shortcoming in the literature. In the future, the development of alloplasts that approximate the ideal implant with low complication rates is warranted. The technology of prefabrication of precise three-dimensional bioactive and biocompatible implants might reduce the incidence of complications and lower the chance of failure.

## Conclusion

To recapitulate, this meta-analysis suggested an acceptable rate of complications and revision surgery with synthetic materials. Synthetic materials might be a proper option when the use of autogenous grafts is not applicable. Judicious case selection and prompt management of complications are crucial whit alloplastic materials. Some practical clinical recommendations may be helpful in future research and clinical procedures. These recommendations are just based on experts’ experience.

## Data Availability

Not applicable.

## Abbreviations

ePTFE: Polytetrafluoroethylene
PRISMA: Preferred Reporting Items for Systematic Reviews
RCT: Randomized clinical trial
CCT: Controlled clinical trial
